# B7-H7 Is Inducible on T Cells to Regulate Their Immune Response and Serves as a Marker for Exhaustion

**DOI:** 10.3389/fimmu.2021.682627

**Published:** 2021-06-01

**Authors:** Khang Luu, Herbert Schwarz, Andreas Lundqvist

**Affiliations:** ^1^ Department of Physiology, Yong Loo Lin School of Medicine, National University of Singapore, Singapore, Singapore; ^2^ NUS Immunology Programme, Life Sciences Institute, National University of Singapore, Singapore, Singapore; ^3^ NUSMED Immunology Translational Research Programme, National University of Singapore, Singapore, Singapore; ^4^ Integrative Sciences and Engineering Programme, National University of Singapore, Singapore, Singapore; ^5^ Department of Oncology-Pathology, Karolinska Institutet, Stockholm, Sweden

**Keywords:** immunology, immune checkpoint, T lymphocyte, exhaustion, immunotherapy

## Abstract

The discovery of immune checkpoints highlights the complexity of T cell signalling during an immune response. Upon activation, T cells express several molecules to regulate their function and to prevent overactivation. B7 homolog 7 (B7-H7) is expressed in tumours and associated with a worse prognosis. However, conflicting data regarding its function suggest that it can be both stimulatory and inhibitory. In this study we report that B7-H7 is also expressed on T cells upon cross-linking of CD3 and CD28 and that additional stimulation *via* CD137 further enhances the expression of B7-H7. B7-H7 is preferentially expressed on exhausted Th1 and Tc1 cells with an impaired secretion of TNF-α and IFN-γ. Blockade of B7-H7 with its natural receptor, recombinant CD28H, enhances T cell proliferation and activation. Thus, B7-H7 represents another target for immunotherapy and a biomarker to select for active effector T cells with relevance for adoptive cell transfer therapy.

## Introduction

B7-H7 is originally discovered as *HHLA2* (Human-endogenous-retroviruses–H Long-terminal-repeat Associating protein 2), in the search for new members of the Immunoglobulin (Ig) superfamily (1). For some time, B7-H7 was confusingly addressed as B7-H5, which is now reserved for VISTA (V-domain Ig suppressor of T cell activation), another member of the Ig superfamily. Beside in humans, B7-H7 is also found in several other species including fish, frog, panda and monkey. Curiously, it is not expressed in laboratory mice and rats (2).

In normal tissue, B7-H7 is expressed mainly in the gastrointestinal track, and the placenta, where immunoregulation is essential (3). On leukocytes, its expression is reported mainly on antigen-presenting cells (monocytes, B lymphocytes, and dendritic cells) (4). B7-H7 has also been reported to be widely expressed on a variety of human cancers (such as breast, lung and melanoma), and is associated with worse disease outcome (3, 5–8).

As the name suggests, B7-H7 shares varying degree of amino acid identity and similarity with other human B7 molecules, such as B7.1/CD80, B7.2/CD86, B7-H1/PD-L1, and B7-H2/PD-L2 (2). For T cells to be optimally activated, they need to receive T-cell receptor stimulation (signal 1), costimulatory signal *via* costimulatory receptors like CD28 (signal 2) and cytokine stimulation (signal 3) (9). B7 family members CD80 and CD86 directly costimulate T cells *via* CD28 (9), while members like PD-L1 and PD-L2 inhibit T cell activation *via* PD-1 (10). Given the essential roles of B7 family molecules, it is reasonable to assume that B7-H7 is involved in the regulation of immune response. At the time B7-H7 function was first assessed, two independent groups showed conflicting results that B7-H7 can be both stimulatory and inhibitory. Zhao et al. reported that B7-H7 inhibit T cells proliferation and cytokine secretion (2), while Zhu et al. demonstrated that B7-H7 co-stimulates T cells *via* its receptor CD28H (4). Following these initial report, more recent studies have also reported both inhibitory and stimulatory functions of B7-H7 (11–13).

Since different groups presented conflicting data on the function of B7-H7, a concept of dual functionality was proposed, as is the case of CD80/CD86 and CD28/CTLA-4. Along these lines, Bhatt et al. recently proposed that CD28H acts as an immunostimulatory receptor whereas KIR3DL3 instead inhibit immune responses upon ligation of B7-H7 (14).

The definition of T cell exhaustion remains a debatable and rapidly developing field in immunology, and there is no single consensus to define T cell exhaustion. During chronic antigen presentation, T cells assume a hypofunctional state with reduced effector functions, cytokine secretion, proliferation, and increased expression of immunoinhibitory checkpoints. This phenomenon is not always viewed as detrimental because it is necessary to prevent immunopathology, limit damage to normal tissue, and allow the T cell to persist and control the disease (15).

In this study, we report that T cells can also express B7-H7 and that the expression is only induced upon activation. T cells expressing B7-H7 also shows an exhausted type 1 T cell phenotype, and blockade of B7-H7 with recombinant human CD28H significantly increases T cell activation and proliferation.

## Materials and Methods

### Gene Expression Data Analysis

For *HHLA2*/B7-H7 mRNA expression analysis in normal immune subsets, we employed In Silico Transcriptomic (IST) Online by Medisapiens (https://ist.medisapiens.com/), which comprises of a large database from normal and pathological human tissues and cells. We also used Human Protein Atlas project (https://www.proteinatlas.org/) (16). Both databases are updated as of 17^th^ Mar 2021.

**Figure 1:**
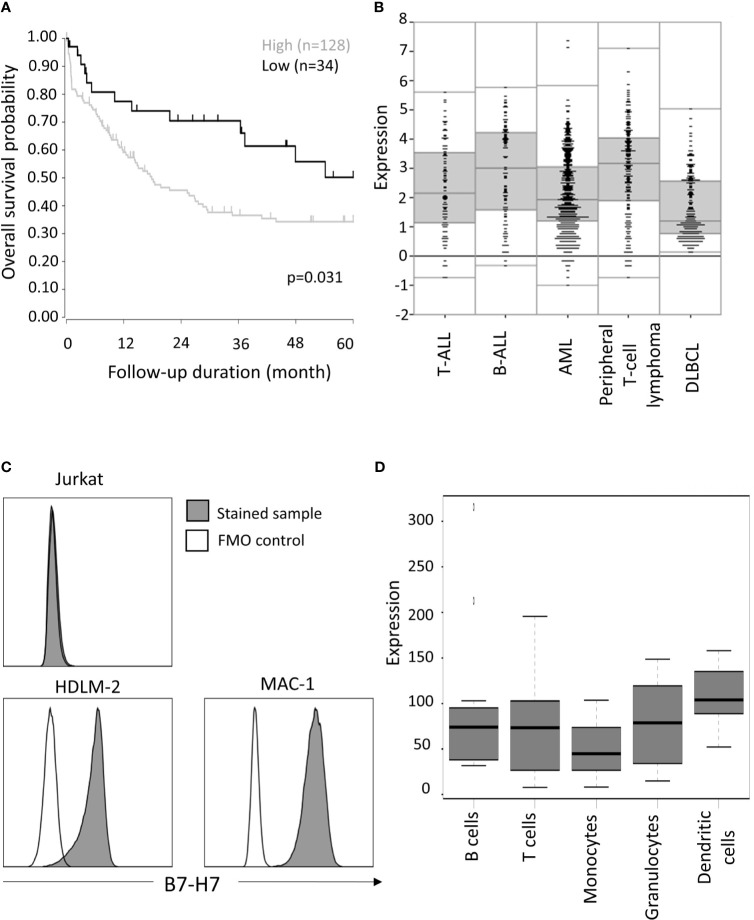
Expression of *HHLA2/*B7-H7 in T cells. **(A)** Overall survival outcome of T-cell peripheral lymphoma patients with high (expression value ≥3) *versus* low (expression value<3) expression level of B7-H7. Of the 193 patients, only 162 provided survival data, for which overall survival probability over five years is calculated, using the log-rank test. **(B)** B7-H7 RNA expression analysis according to R2 data visualization platform for DLBCL (GEO: gse87371), B-ALL (GEO: gse7440), AML (GEO: gse14468) T-ALL (GEO: gse10609) and T-cell peripheral lymphoma patients (GEO: gse58445). **(C)** B7-H7 expression on different cell lines with T cells origin: Jurkat, HDLM-2 and MAC-1. Cells were gated for live and single cells. **(D)** B7-H7 RNA expression analysis on normal immune cell subsets, according to IST Online MediSapiens.

Published databases were extracted and visualized *via* the R2: Genomics Analysis and Visualization Platform (http://r2.amc.nl). For **Figure 1A**, T-cell peripheral lymphoma patient overall survival probability analysis, out of 193 patients, only 162 patients with survival data were included, the follow up period was selected to be 60 months (GEO: gse58445) (17).

For mRNA expression visualization in R2, the following databases were used: B-ALL (GEO: gse7440, n=98) (18), DLBCL (GEO: gse87371, n=223) (19), AML (GEO: gse14468, n=525) (20), T-ALL (GEO: gse10609, n=92) (21), T-cell peripheral lymphoma (GEO: gse58445, n=193) (17).

### Cell Lines

HDLM-2 (kindly provided by Dr. G Rassidakis, Karolinska Institutet) was cultured at 37°C in 5% CO_2_, in RPMI 1640 medium (Gibco, Thermo Fisher Scientific), supplemented with 20% heat inactivated fetal bovine serum (FBS) (Gibco), and 1% Penicillin and Streptomycin (Gibco). Jurkat (ATCC) and MAC-1 cells (kindly provided by Dr. G Rassidakis, Karolinska Institutet) were cultured at 37°C with 5% CO_2_, in RPMI 1640 medium (Gibco), supplemented with 10% heat inactivated FBS (Gibco), and 1% Penicillin and Streptomycin (Gibco).

### Primary Human Cell Isolation

Peripheral blood mononuclear cells (PBMC) were isolated from buffy coat by Ficoll gradient centrifugation (GE healthcare) and washed three times in PBS. T cells were negatively selected with magnetic-assisted cell sorting (MACS) from PBMC with the use of CD8^+^ T cell isolation kit or Pan T cell isolation kit (Miltenyi Biotec). Primary human T cells were cultured in RPMI 1640 medium (Gibco), supplemented with 10% heat inactivated FBS (Gibco), and 1% Penicillin and Streptomycin (Gibco). Monocytes were positively selected with MACS by CD14 Microbeads (Miltenyi Biotec) and were cultured in RPMI 1640 medium (Gibco), supplemented with 10% heat inactivated FBS (Gibco), and 1% Penicillin and Streptomycin mixture (Gibco).

### Activation of Human Primary Cells

For T cell activation, T cells were cultured at a concentration of 10^6^ cells/ml. TransAct™ (Miltenyi Biotec), a nanomatrix coated with CD3 agonist and CD28 agonist, was used at the manufacturer’s recommended dilution of 1:100, unless otherwise stated. When used, Torin-1 (Selleck Chemicals) was added to cultures at 1 nM. To study the dynamic of B7-H7 expression, T cells were treated with TransAct™ for 72 hours, washed and reseeded in fresh medium containing 100 IU/ml of IL-2 (Novartis) or fresh medium containing the recommended dose of TransAct™ for another 72 hours. For blocking of B7-H7, recombinant human CD28H (R&D systems) was added at 5 µg/ml together with suboptimal dose of TransAct™ (1:1000), and the cells were cultured for 72 to 96 hours. To immobilize CD137L or anti-CD3, recombinant human CD137L (R&D systems) or anti-CD3 (clone OKT3, Biolegend) was coated at 1 µg/ml on a Falcon^®^ 96-well Clear Flat Bottom (Corning Life Sciences) overnight at 4°C, then washed twice with PBS, before T cells were seeded. 100 ng/mL Phorbol 12-myristate 13-acetate (PMA) and 1 μg/mL ionomycin was added to purified T cells for 24 hours.

### Mixed Lymphocyte Reaction

Isolated monocytes were seeded at 10^6^ cells/ml, supplemented with 100 ng/ml IL-4 at and 80 ng/ml GM-CSF (Peprotech) for five days, to be differentiated to monocyte-derived dendritic cells. For maturation of DC, LPS (SigmaAldrich) was added on day five at 5 µg/ml. On day six, mature DC were harvested, and 4 x 10^4^ mature DC were co-cultured with 2 x 10^5^ allogenic T cells for six days.

### Flow Cytometry

For surface marker staining, cells were incubated with dead cell marker, followed by Fc receptor blocking reagents, then with a mixture containing the relevant fluorophore-conjugated antibodies (**Supplementary Table 1**), and finally using a fixing reagent, BD Cytofix™ (BD Bioscience). For intracellular staining, eBioscience™ Foxp3/Transcription Factor Staining Buffer Set (Thermo Fisher Scientific) was used according to the manufacturer’s instructions. All flow cytometry data was analyzed by Treestar FlowJo program.

### Statistical Analysis

Statistical analyses were performed using Prism Graphpad software. Survival analysis was performed using the R2 platform. Student’s t test was used to compare the difference between two groups, and one-way ANOVA with multiple comparison tests were used to compare multiple groups. Overall survival probability was calculated using the log-rank test.

## Results

### B7-H7 Is Expressed on Human T-Cell Haematological Cancers and Cell Lines of T- Cell Origin

Consistent with previous reports that B7-H7 expression is associated with poor prognosis, we observed that high B7-H7 expression was a prognosis marker for peripheral T-cell lymphoma (**Figure 1A**). Comparing B7-H7 expression level of peripheral T-cell lymphoma patients with that of other haematological cancers, similar levels of expression was observed for B-cell acute lymphoblastic leukaemia (B-ALL) and T-cell acute lymphoblastic leukaemia (T-ALL) whereas Diffuse Large B Cell Lymphoma (DLBCL) and acute myeloid leukaemia (AML) showed lower expression of B7-H7. (**Figure 1B**). To validate these results, B7-H7 protein expression was assessed on the thymic T cell line Jurkat, HDLM-2 (A Hodgkin lymphoma cell line with T cell origin (22)), and MAC-1 (a cutaneous T-cell non-Hodgkin lymphoma cell line). While no expression of B7-H7 was detected on Jurkat cells, both HDLM-2 and MAC-1 expressed high levels of B7-H7 (**Figure 1C**).

While antigen-presenting cells and myeloid cells were reported to be the major cell types that expressed B7-H7, mRNA expression data from IST Medisapiens demonstrated that B7-H7 was expressed on most immune cells at the RNA level, including T cells (**Figure 1D**). Other public databases also verified that while T cell might not have the highest level of B7-H7 expression, B7-H7 transcript was consistently detected in T cells (**Figure S1**). Taken together, these findings suggest that primary T cells and T-cell related tumours could express B7-H7 at the protein level.

### B7-H7 Expression Is Induced on T Cells Upon Activation *via* CD3 and CD28

**Figure 2:**
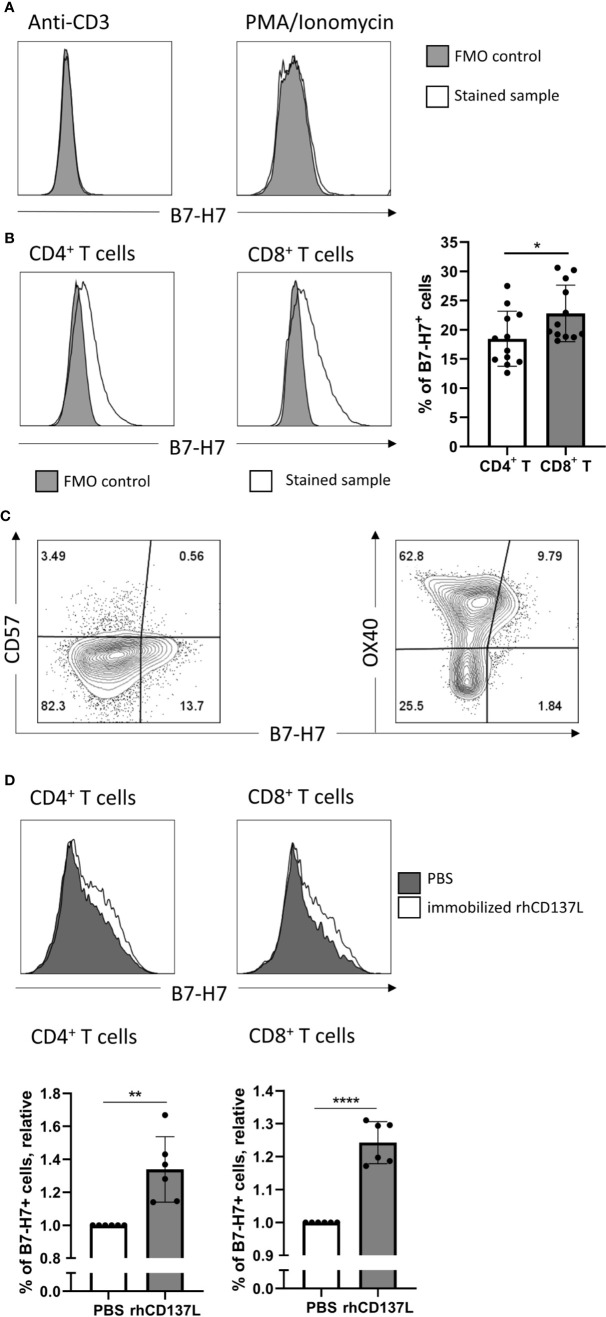
Induction of B7-H7 expression on T cells upon activation. **(A)** Expression of B7-H7 in T cells activated with CD3 crosslinking or PMA/ionomycin for 72 and 24 hours respectively, n=2. **(B)** Expression of B7-H7 in CD4^+^ and CD8^+^ T cells upon crosslinking of CD3 and CD28 for 72 hours, n=12. **(C)** Expression of B7-H7, CD57, and OX40 in total T cells upon crosslinking of CD3 and CD28 for 48 hours, n=4. **(D)** Expression of B7-H7 of TransAct™ activated T cells in the presence or absence of rhCD137L, n=6. Cells were gated for live and single cells, *p < 0.05, **p < 0.01, ****p < 0.0001, unpaired Student’s T test.

**Figure 3:**
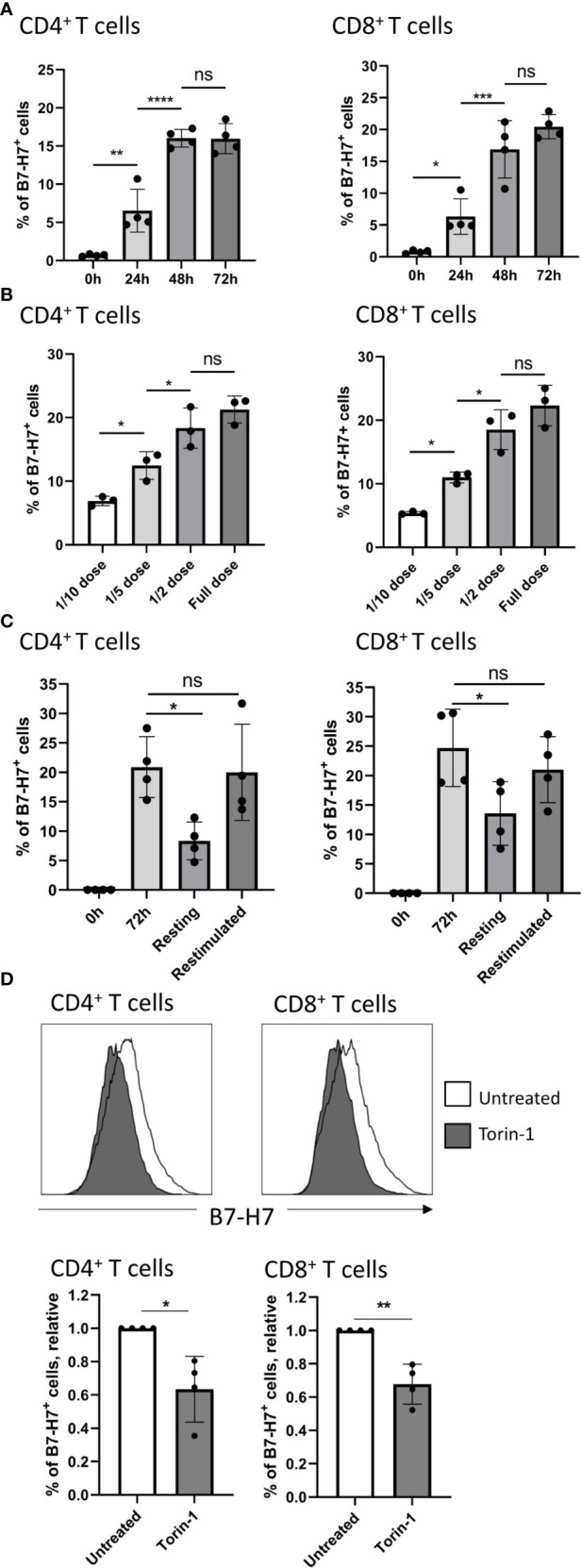
Expression of B7-H7 is dynamic and dependent on mTOR activity. **(A)** B7-H7 expression on CD4+ and CD8+ T cells at 24, 48, or 72 hours, n=4. **(B)** Expression of B7-H7 at different dilutions of TransAct™. Full dose of TransAct™ is 1:100 dilution, n=3. **(C)** B7-H7 expression in activated, resting and re-stimulated T cells. Purified T cells were activated with the recommended dose of TransAct™ (1:100) for 72 hours, washed and reseeded in fresh medium containing 100 IU/ml of IL-2 (‘resting’) or fresh medium containing the standard dose of TransAct™ for another 72 hours (‘restimulated’), n=4. **(D)** Relative expression of B7-H7 in TransAct™ (1:100) activated T cells in the presence or absence of the mTOR inhibitor Torin-1 (1nM) or untreated control, n=4. Cells were gated for live and single cells. ns, not significant, *p < 0.05, **p < 0.01, ***p < 0.001 ****p < 0.0001. For comparison between 2 variables, unpaired Student’s T test was used. To compare multiple variables, one-way ANOVA was used, followed by multiple comparison tests.

Consistent with previous reports, the expression of B7-H7 was absent on resting T cells (**Figure 3A**). Cross-linking of CD3 alone or mitogenic stimulation by Phorbol 12-myristate 13-acetate (PMA) and Ionomycin did not increase B7-H7 expression (**Figure 2A**). However, upon activation of purified T cells through cross-linking of CD3 and CD28, both CD4^+^ and CD8^+^ T cells expressed B7-H7, demonstrating that other signals including the cross-linking of CD28 was needed for the induction of B7-H7 expression. Although significant, the expression of B7-H7 was on average 4% higher in CD8^+^ T cells compared with CD4^+^ T cells (**Figure 2B**). Analysis of markers of T cell differentiation revealed that the majority of the B7-H7^+^ T cells are CD57^-^ and OX40^+^, describing a state of activation but not terminal differentiation (**Figure 2C**).

To test if additional costimulatory signals such as CD137 stimulation would further increase the expression of B7-H7, CD3/CD28 activated T cells were cultured in the presence or absence of recombinant human CD137 ligand (rhCD137L). Indeed, the expression of B7-H7 was significantly higher in both CD4^+^ and CD8^+^ T cells in the presence of rhCD137L (**Figure 2D**). To test whether other stimuli can induce B7-H7 on T cells, we performed a mixed lymphocyte reaction and observed B7-H7 on activated CD25^+^ T cells (**Figure S2**).

### The Expression of B7-H7 Is Dynamic and Dependent on the Stimulation Strength and mTOR Activity

Upon further investigation into the expression kinetics, B7-H7 was detected at 24 hours after stimulation, and the expression plateaued after 72 hours, on both CD4^+^ and CD8^+^ T cells (**Figure 3A**). To test whether the strength of CD3/CD28 cross-linking influences the expression of B7-H7, increasing concentrations of CD3/CD28 agonists (TransAct™) were added to T cells. At lower concentrations, the expression of B7-H7 was significantly reduced on both CD4^+^ and CD8^+^ T cells (**Figure 3B**). To further understand if the expression of B7-H7 fluctuates during activation, T cells were first activated with agonists for CD3 and CD28 (TransAct™) for 72 hours and thereafter washed and rested in media containing IL-2 for maintenance, or immediately restimulated with CD3 and CD28 agonists (TransAct™) for another 72 hours. In restimulated CD4^+^ and CD8^+^ T cells, the expression of B7-H7 was maintained, whereas it was significantly reduced in rested CD4^+^ and CD8^+^ T cells (**Figure 3C**). Since mTOR activity can affect the expression of immune checkpoints, including PD-1 and PD-L1 (23–25), mTOR was inhibited during the activation of T cells to test if its activity is linked to the expression of B7-H7. Indeed, in the presence of the mTOR inhibitor Torin-1, the expression of B7-H7 is significantly reduced both CD4^+^ and CD8^+^ T cells, demonstrating that the expression of B7-H7 is dependent on the mTOR pathway (**Figure 3D**).

### T Cells Use B7-H7 to Regulate Their Immune Response

**Figure 4:**
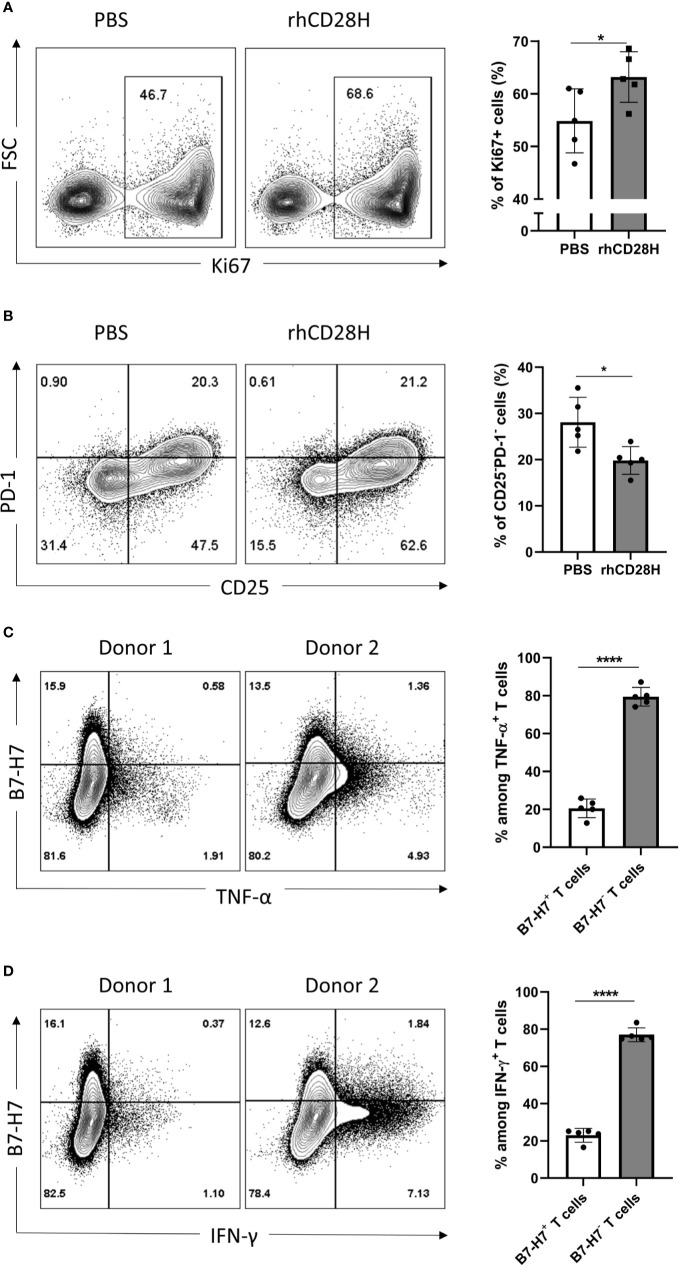
Blocking B7-H7 enhances T cell proliferation. Purified T cells were activated with suboptimal 1/10 TransAct™ dose (1:1000) for three to four days in the presence of PBS or 5 µg/ml of recombinant human CD28H (rhCD28H) and stained for **(A)** ki67 or **(B)** CD25 and PD1, n=5. Purified T cells were activated with recommended dose of TransAct™ (1:100) for three days and stained for B7-H7, **(C)** TNF-α and **(D)** IFN-γ, n=5. Dot plots from two representative donors are shown. Cells were gated for live and single cells. *p < 0.05, ****p < 0.0001, unpaired Student’s T test.

Given the expression of B7-H7 on activated T cells, we hypothesized that it might act as a negative feedback mechanism to regulate T cell activation. Thus, B7-H7 was blocked with soluble recombinant human CD28H (rhCD28H) as it was identified as one of B7-H7’s receptors. Indeed, in the presence of rhCD28H, proliferation of T cells was significantly increased (**Figure 4A**). Furthermore, the frequency of non-activated CD25^-^PD-1^-^ T cells was significantly lower in the presence of rhCD28H (**Figure 4B**). We also observed a consistent, albeit statistically insignificant, upregulation of IFN-γ secretion upon the addition of rhCD28H (data not shown). Analysis of B7-H7 expression in activated T cells revealed that B7-H7^-^ T cells produced higher levels of inflammatory cytokines such as TNF-α and IFN-γ (**Figures 4C, D**). These results support that B7-H7 on T cells functions as an inhibitory ligand to control T cell activation, and T cells that express B7-H7 do not produce the inflammatory cytokines TNF-α and IFN-γ.

### B7-H7 as a Marker for Exhausted Th1 and Tc1 Subsets

Since B7-H7^+^ T cells secrete minimal amounts of TNF-α and IFN-γ, we hypothesized that they were either exhausted or that they did not belong to the Th1 or Tc1 subpopulations. The phenotypic markers CXCR3 and CCR6 were previously described to distinguish cytotoxic and helper T cell subpopulations (26–28). Thus, to investigate for different subsets of T cells in relation to B7-H7 expression, activated T cells were analyzed for the expression of CXCR3 (a marker of type-1 phenotype) and CCR6 (a marker of type-17 phenotype). When all T cells were assessed, type-1 cells were assessed regardless of whether the T cell expressed CD4 or CD8. Hence, the term Th1/Tc1 refers to both subpopulations. Whenever the analysis could tell that those T cells express CD4 or CD8, the term Th1 or Tc1 was used respectively.

**Figure 5:**
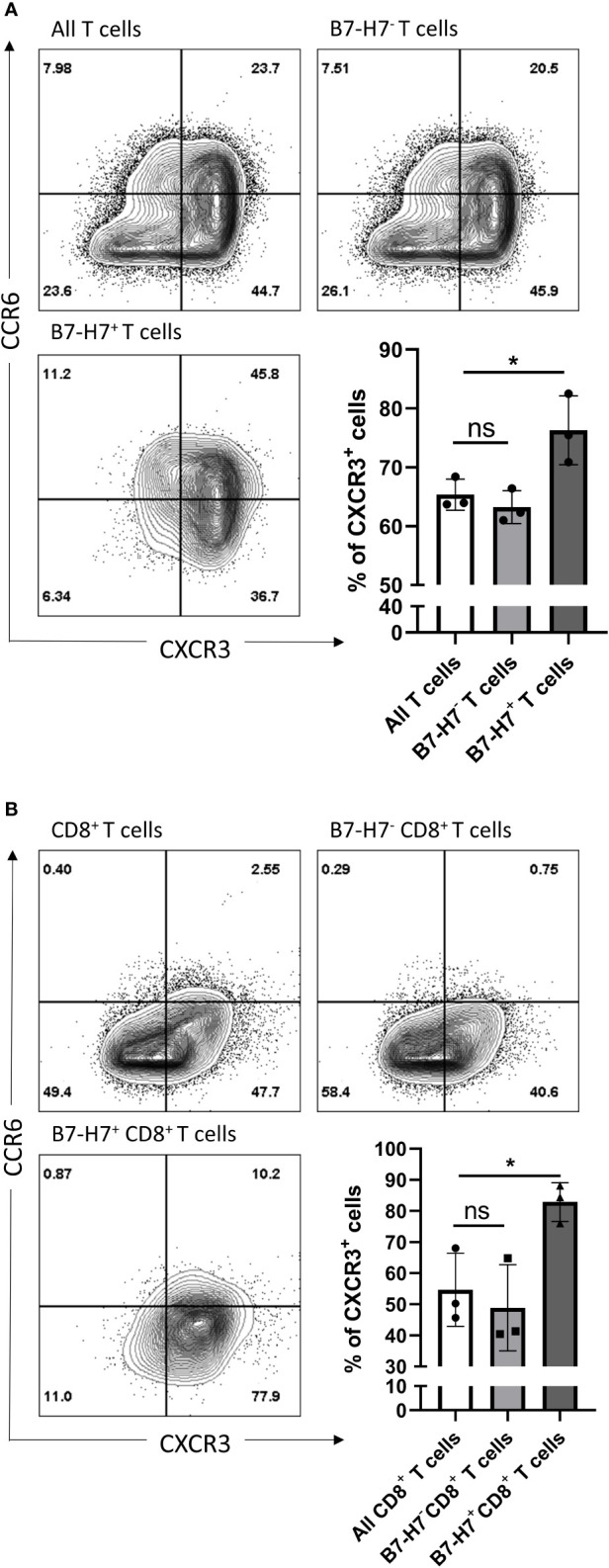
CXCR3 and CCR6 expression profiles of total and CD8 purified T cells. Purified T cells **(A)** or purified CD8^+^ T cells **(B)** were activated with recommended dose of TransAct™ (1:100) for three days, and assessed for B7-H7, CXCR3 and CCR6, n=3. Cells were gated for live and single cells. ns, not significant, *p < 0.05, one-way ANOVA was used, followed by multiple comparison tests.

Regardless of B7-H7 expression, the majority of T cells committed to the Th1/Tc1 subset upon crosslinking of CD3 and CD28 as evidenced by the high expression of CXCR3 (**Figure 5A**). Furthermore, while the expression profile on B7-H7^-^ T cells closely mirrored that of total T cell population, B7-H7^+^ T cells were enriched in CXCR3 expression, suggesting a stronger type 1 phenotype in B7-H7^+^ T cells (**Figure 5A**).

Upon further subpopulation analysis, this enrichment effect of B7-H7 status for type 1 T cell phenotype was observed in both CD4^+^ and CD4^-^ T cells (**Figure S3**). Notably, the CXCR3 enrichment effect of B7-H7 in CD4^-^ B7-H7^+^ T cells was stronger than in CD4^+^ B7-H7^+^ T cells (**Figure S3B**). Thus, isolated CD8^+^ T cells were used to determine if the enrichment effect still could be clearly observed, as seen on CD4^-^ T cells when whole T cell population was used. CD8^+^ T cells were purified, activated, and analyzed for the expression of B7-H7, CXCR3, and CCR6. Consistent with the subpopulation analysis with CD4^-^ cells and similar to the observation for the total T cell population, the expression profile of CD8^+^ B7-H7^-^ T cells was similar to that of all CD8^+^ T cells, while B7-H7^+^CD8^+^ T cells expressed significantly higher levels of CXCR3 compared with CD8^+^B7-H7^-^ T cells (**Figure 5B**).

**Figure 6:**
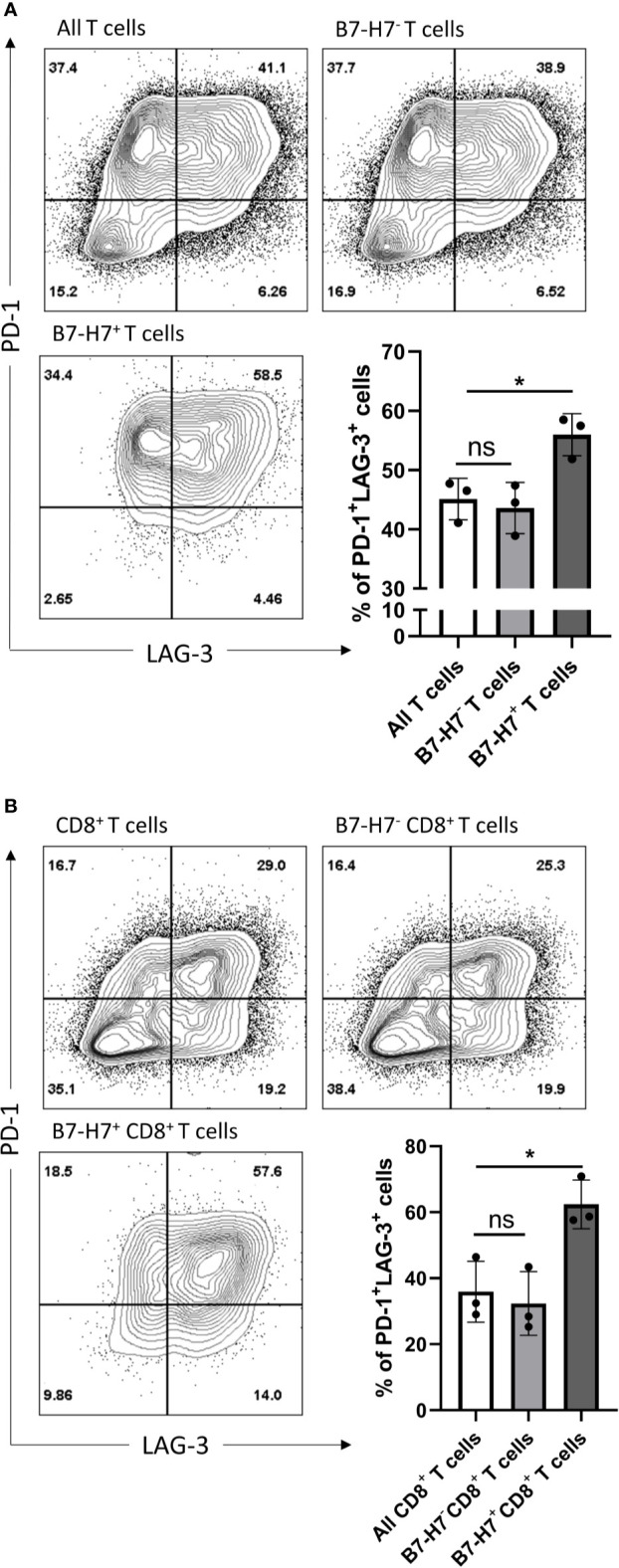
B7-H7^+^ T cells show an exhausted phenotype. Purified T cells **(A)** or purified CD8^+^ T cells **(B)** were activated with recommended dose of TransAct™ (1:100) for three days and assessed for B7-H7, PD-1 and LAG-3, n=3. Cells were gated for live and single cells. ns, not significant, *p < 0.05, one-way ANOVA was used, followed by multiple comparison tests.

Since the majority of B7-H7^+^ T cells expressed markers associated with Th1 or Tc1 subsets and they had an impaired production of IFN-γ and TNF-α, T cells were analyzed for the expression of markers associated with exhaustion. Compared with total T cells, B7-H7^+^ T cells were enriched for PD-1 and LAG-3 (**Figure 6A**). Similarly, when purified CD8^+^ T cells were activated, CD8^+^ B7-H7^+^ T cells also showed significantly higher frequency of PD-1^+^ LAG-3^+^ cells compared with CD8^+^ B7-H7^-^ T cells or total purified CD8^+^ T cells (**Figure 6B**). Taken together, these results show that B7-H7 can serve as a marker for exhausted Th1 or Tc1 subsets that secrete minimal TNF-α or IFN-γ.

## Discussion

As the case frequently is, tumours escape immunosurveillance not by devising a new pathway, but rather by hijacking an existent immunoregulatory pathway. In this study, we report that human primary T cells express B7-H7 upon activation which act as a regulatory mechanism to control T cell activation. The notion that T cells express both the immune checkpoint molecule and its ligand is not unusual. For instance, T cells have been reported to express both PD-L1 and PD-L2, in addition to PD-1 (29, 30). B7-H7 is a new member of that group of ligands. The role of B7-H7 in T cell activation remains controversial. Several studies have shown that ligation of B7-H7 can either stimulate or inhibit T cell responses (2, 4). In analogy to B7.1 and B7.2 that ligate CTLA-4 and CD28 to inhibit and stimulate T cell responses respectively, B7-H7 has been shown to ligate KIR3DL3 and CD28H to inhibit and stimulate T cell responses respectively (14). However, Rieder et al. showed that the inhibitory effect of B7-H7 signalling is evident as early as 24 h after the initial stimulation (11). Nevertheless, KIR3DL3 is not expressed on resting T cells and the expression is less than 5% upon cross-linking CD3 and CD28 (14). It is therefore plausible that other inhibitory receptors that mediate immune regulation by B7-H7 exist.

In this study, we demonstrate that recombinant CD28H may block B7-H7 and enhance the immune response. However, recombinant CD28H may not be the most optimal agent to block B7-H7 because it may leave other regions of B7-H7 available for binding to inhibitory receptor(s). A specific antibody that preferentially blocks the interaction between B7-H7 and its inhibitory receptor(s), rather than its stimulatory receptor, would likely be more beneficial as an immunotherapy. Currently, there is no commercially available agent that can specifically block the interaction between B7-H7 and immunoinhibitory receptors, while leaving immunostimulatory receptor alone. Rather than blocking B7-H7, which can costimulate T cells *via* CD28H, future developments should focus on blocking its inhibitory receptor(s). To get there, the identification of these inhibitory receptor(s) is essential.

Consistent with the findings of Zhao et al., immobilised anti-CD3 antibody alone did not induce B7-H7 expression on T cells (2). Furthermore, no induction of B7-H7 was observed upon mitogenic stimulation with PMA and ionomycin. Only when cross-linking CD3 and CD28 or in a mixed lymphocyte reaction, the expression of B7-H7 was significantly increased. It has also been reported before that agonistic anti-CD3 antibody alone or PMA-ionomycin treatment can induce T cells to produce IL-2 (31, 32). Thus, IL-2 alone is not sufficient to induce B7-H7 expression and cannot substitute CD28 signal. However, it cannot be discounted that IL-2 can enhance B7-H7 expression on T cells once B7-H7 is induced. To formally conclude that IL-2 is essential, neutralizing antibodies to IL-2 would have been needed to include in the assays. Thus, further investigation is required to assess the effect of various T cell-secreted cytokines on B7-H7 expression.

The expression of B7-H7 on T cells has previously been shown at the mRNA level, since Aznar et al. found that agonistic anti-CD137 antibody enhance B7-H7 mRNA expression, in addition to agonistic anti-CD3 antibody treatment (33). In our hand, the protein expression of B7-H7 did increase in the presence of recombinant human CD137 ligand stimulation in T cells upon crosslinking CD3 and CD28. These findings demonstrate that the costimulatory signal *via* CD28/CD137 is essential for B7-H7 to be expressed at protein level on T cells.

Our data suggests that B7-H7 is preferentially expressed on CD8^+^ T cells upon activation. Similarly, a recent study in orthotopic KPC tumours showed that the expression of PD-L1 (B7-H1) was expressed in approximately 40% and 60% of CD4^+^ and CD8^+^ T cells respectively (30). Although there is no clear explanation why CD8^+^ T cells would express higher levels of immune checkpoint ligands, we speculate that it may provide a faster and stronger negative feedback to inhibit CD8 T cell cytotoxic responses.

Our results identify B7-H7 as a marker of a subset of Th1 or Tc1 cells that do not secrete TNF-α and IFN-γ. The fact that CD57 and B7-H7 are not expressed on the same T cell suggest that B7-H7^+^ T cells are not senescent or terminally differentiated. Our results show that crosslinking of CD3 and CD28 induces most T cells to express CXCR3, a marker of type 1 polarization (Th1/Tc1). This is consistent with previous reports showing that CD3 and CD28 activation favours the generation of Th1 and Tc1 T cells (34, 35). Furthermore, B7-H7 allows us to better identify CXCR3^+^ T cells, as seen by the enrichment of CXCR3^+^ T cells among the B7-H7^+^ T cells. Thus, it is surprising to find that B7-H7^+^ T cells are poor producers of TNF-α and IFN-γ.

Upon further investigation, we found that B7-H7^+^ T cells are mostly exhausted, as evidenced by their PD-1 and LAG-3 expression level. We also observed that B7-H7^+^ T cells express a higher level of CTLA-4 and TIM-3 (**Figure S5**). The overexpression of inhibitory receptors including PD-1 and LAG-3 have been established as markers of T cells exhaustion (36), and that T cells co-expressing both PD-1 and LAG-3 are significantly more exhausted than T cells that express either PD-1 or LAG-3 alone (37). Furthermore, PD-1 and LAG-3 has been described to cooperate to maintain functional T cell exhaustion (38). In this study, we demonstrate that B7-H7 can be used to identify exhausted T cells since they not only secrete low levels of inflammatory cytokines but also express higher levels of both PD-1 and LAG-3. Since PD-1 can also be expressed on resting T cells, B7-H7 may be better positioned as a late-stage marker of T cell exhaustion since it is only expressed upon activation (39). Collectively, we have identified that exhausted T cells express B7-H7 and that blockade of B7-H7 improves the proliferation of T cells. While our data does not examine every aspects of T cell exhaustion, we managed to show decreased secretion of cytokines, as well as co-expression of other immune checkpoints in B7-H7^+^ T cells. This fits in the broad definition of T cell exhaustion and may introduce B7-H7 into the group of surface markers that define exhausted T cells.

The distinction between an activation marker and an exhaustion marker is not always clear. The same doubt can be applied for PD-1 and CTLA-4. Both PD-1 and CTLA-4 are rapidly induced on T cells after activation (40, 41). As B7-H7 is only induced on activated T cells, it can also be considered a marker of T cells activation. However, not every activated T cell expresses B7-H7 and those that do have less cytokine secretion. Hence, we view B7-H7 as an exhaustion marker rather than an activation marker.

As T cells that express B7-H7 limit the activation of bystander T cells and they themselves do not contribute inflammatory cytokine secretion, a potential application of this finding is that selecting for B7-H7^-^ T cells can be considered to improve the efficacy of adoptive T cell therapy. During a chronic infection, exhausted T cells may express high levels of B7-H7. Whether these T cells should be targeted remains debatable. Immunoregulation *via* B7-H7 in particular, and T cell exhaustion in general, may not be inherently good or bad. For instance, T cell exhaustion is important and evolutionarily conserved to limit autoimmunity, prevent damage to nearby tissues and allow T cell to survive to contain the infection (15). Further studies are required to explore the impact of targeting B7-H7 during a chronic infection.

Furthermore, it would be interesting to explore the role of B7-H7 expression on intratumoural T cells to regulate immune responses. Since tumour and chronic infection are the main causes for T cell exhaustion, it is plausible that intratumoural T cells have upregulated B7-H7 expression. Further investigation into the different stimuli that induce B7-H7 on T cells may allow us to limit the negative influence of this pathway on immunotherapies. Additionally, it is vital to identify immunoinhibitory receptors for B7-H7. Since the B7-H7 – CD28H interaction may be beneficial for the immune response, it is preferable to block those immunoinhibitory receptors, rather than B7-H7 itself.

In the case of autoimmunity, since many autoimmune conditions are characterised by abnormal T cell function, it is worth exploring the expression of B7-H7 on T cells. Autoreactive T cells, being continuously activated, may express B7-H7. For those conditions where T cells express high B7-H7, it may be logical to block CD28H, while allowing B7-H7 to bind to other immunoinhibitory receptors.

## Data Availability Statement

The original contributions presented in the study are included in the article/**Supplementary Material**. Further inquiries can be directed to the corresponding author.

## Authors Contributions

KL performed the experiments, analysed the data and wrote the manuscript. HS and AL supervised the study and wrote the manuscript. All authors contributed to the article and approved the submitted version.

## Funding

The study was funded by The Swedish Cancer Society (#CAN 2018/451) and The Cancer Research Foundations of Radiumhemmet (#181183).

## Conflict of Interest

The authors declare that the research was conducted in the absence of any commercial or financial relationships that could be construed as a potential conflict of interest.

## Supplementary Material

Supplementary Table 1 List of staining reagents used for flow cytometry assay.
