# Supplementary material for: B7-H7 Is Inducible on T Cells to Regulate Their Immune Response and Serves as a Marker for Exhaustion
Source: Front Immunol. 2021 Jun 1;12:682627. doi: 10.3389/fimmu.2021.682627 (PMC8205074; doi:10.3389/fimmu.2021.682627)
Supplement: Supplementary Table 1 — List of staining reagents used for flow cytometry assay. [file Table_1.docx]

| **Target/Designation** | **Fluorophore** | **Clone** | **Catalog number** | **Supplier** |
| --- | --- | --- | --- | --- |
| AQUA fixable dead cell stain | Not applicable | Not applicable | L34957 | ThermoFisher |
| Violet fixable dead cell stain | Not applicable | Not applicable | L34955 |  |
| Near IR fixable dead cell stain | Not applicable | Not applicable | L10119 |  |
| B7-H7 | PE-CY7 | MA57YW | 25-6537-42 | ThermoFisher |
| CD3 | FITC | UCHT1 | 300406 | Biolegend |
| CD4 | PE | RPA-T4 | 555347 | BD Biosciences |
| CD4 | Pacific Blue | RPA-T4 | 558116 | BD Biosciences |
| CD8 | FITC | RPA-T8 | 555366 | BD Biosciences |
| CD25 | PE | M-A25 | 356104 | Biolegend |
| CD57 | Pacific Blue | HCD57 | 322316 | Biolegend |
| CCR6 | PE | 53103 | FAB195P | R&D |
| CXCR3 | AF647 | TG1/CXCR3 | 334903 | Biolegend |
| LAG-3 | FITC | 11C3C65 | 369307 | Biolegend |
| OX40 | AF647 | ACT35 | 350017 | Biolegend |
| PD-1 | APC | MIH-4 | 17-9969-41 | ThermoFisher |
| IFN-γ | FITC | 4S.B3 | 552882 | BD Biosciences |
| TNF-α | FITC | MAb11 | 502906 | Biolegend |
| Ki67 | AF647 | B56 | 558615 | BD Biosciences |
| CTLA-4 | APC | L3D10 | 349907 | Biolegend |
| TIM-3 | BV421 | F38-2E2 | 345007 | Biolegend |
