# Supplementary figures and images for: B7-H7 Is Inducible on T Cells to Regulate Their Immune Response and Serves as a Marker for Exhaustion
Source: Front Immunol. 2021 Jun 1;12:682627. doi: 10.3389/fimmu.2021.682627 (PMC8205074; doi:10.3389/fimmu.2021.682627)

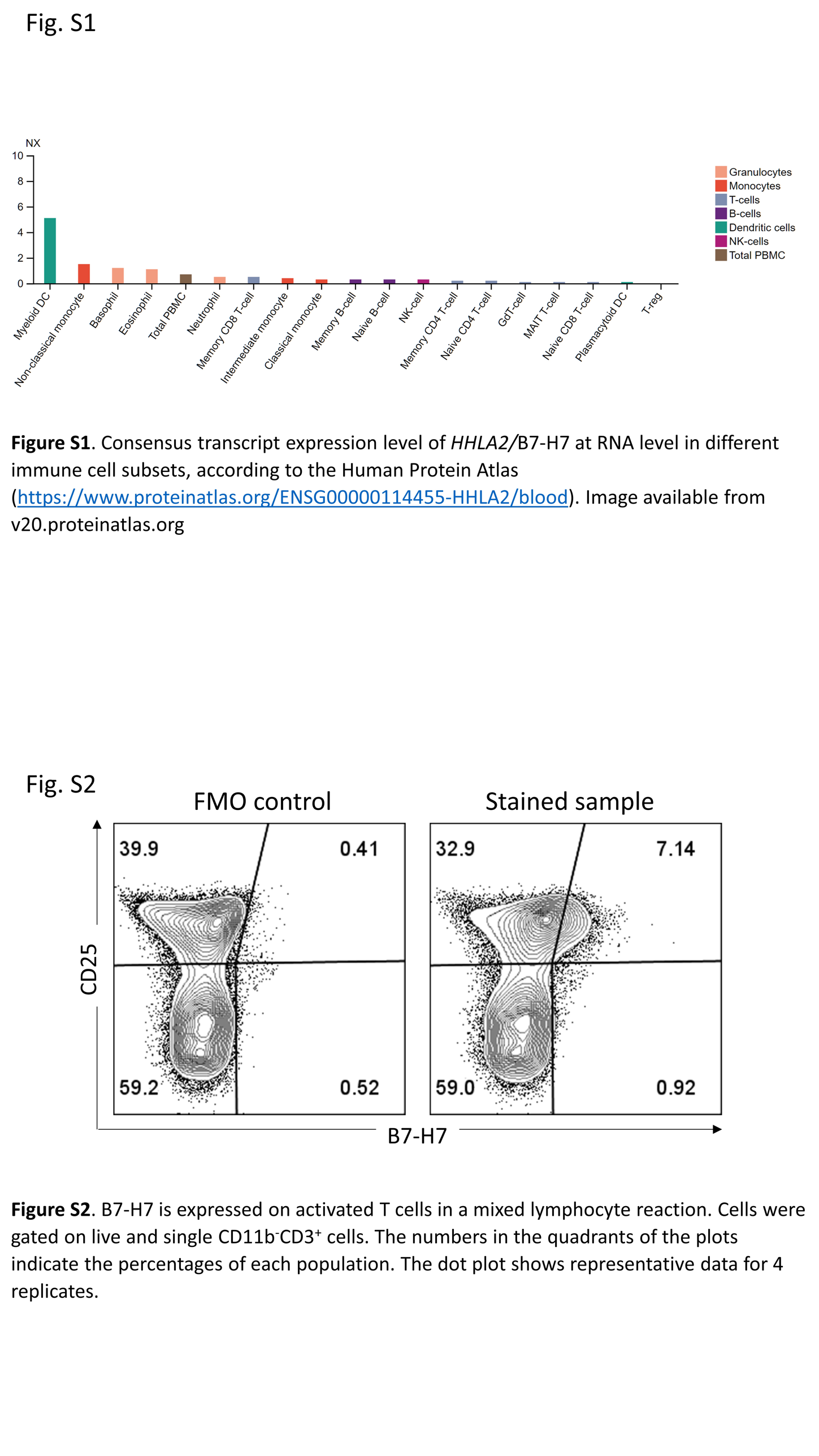

Supplement: Supplementary file 2 [file Image_1.jpeg]

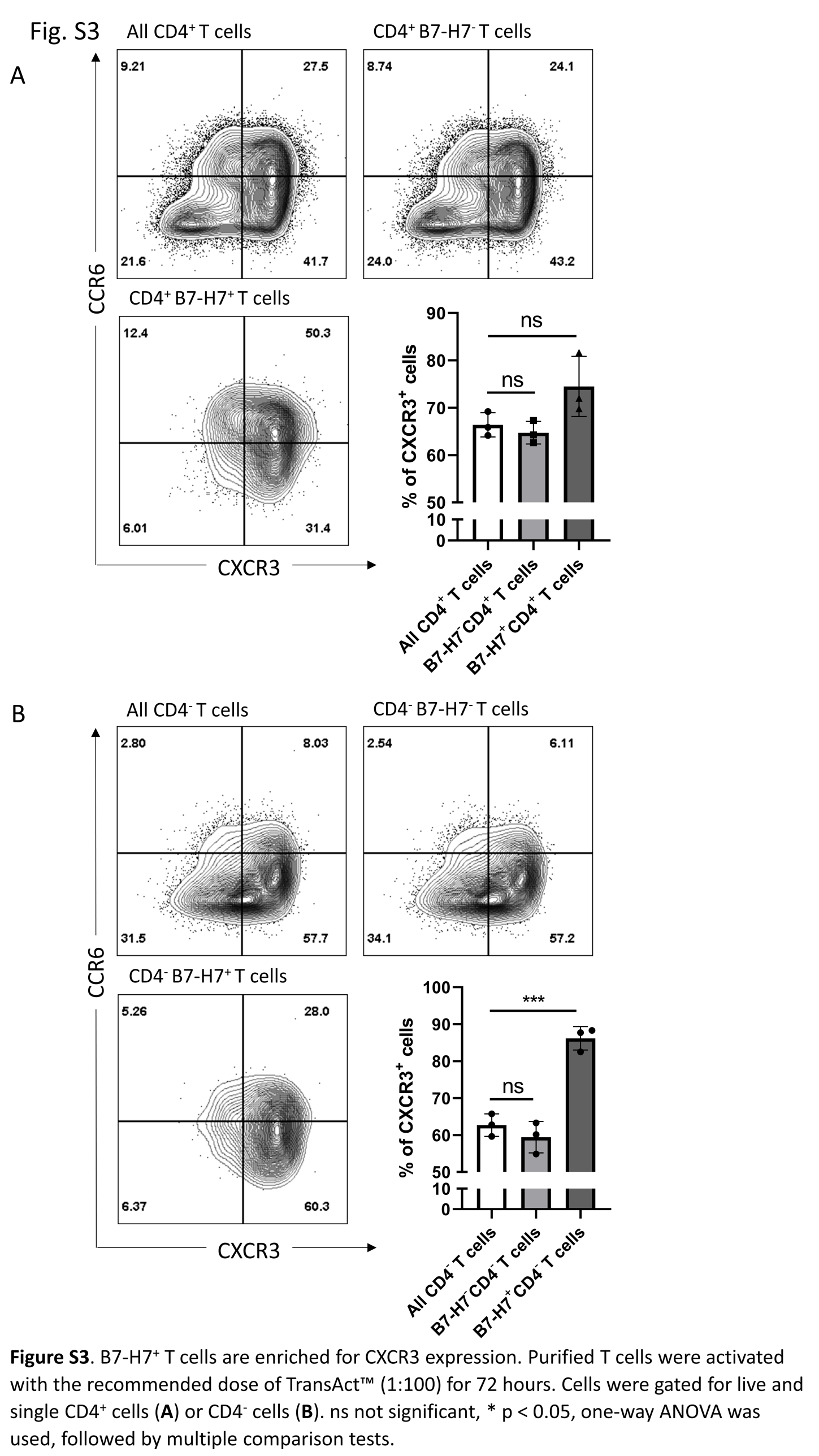

Supplement: Supplementary file 3 [file Image_2.jpeg]

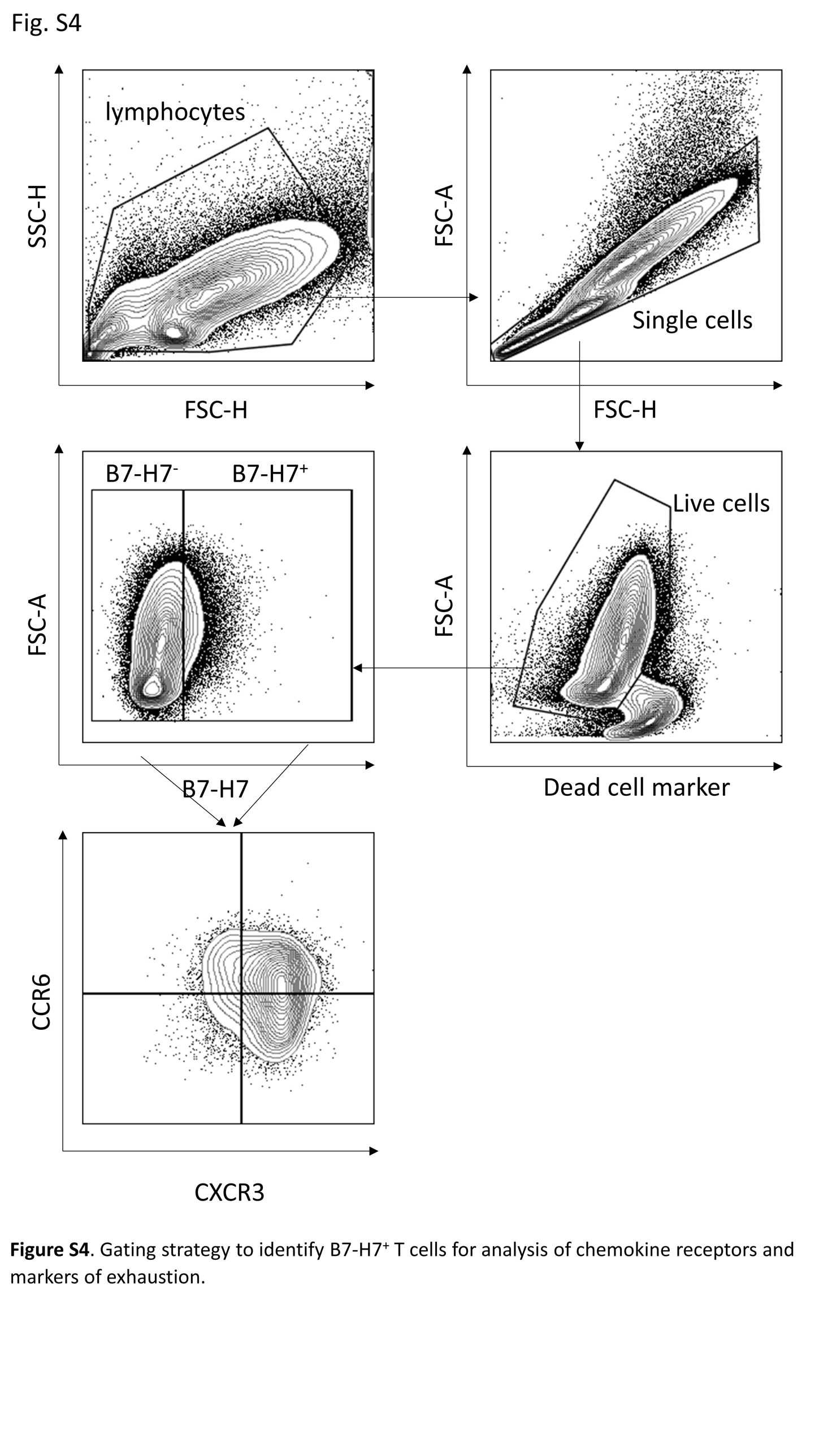

Supplement: Supplementary file 4 [file Image_3.jpeg]

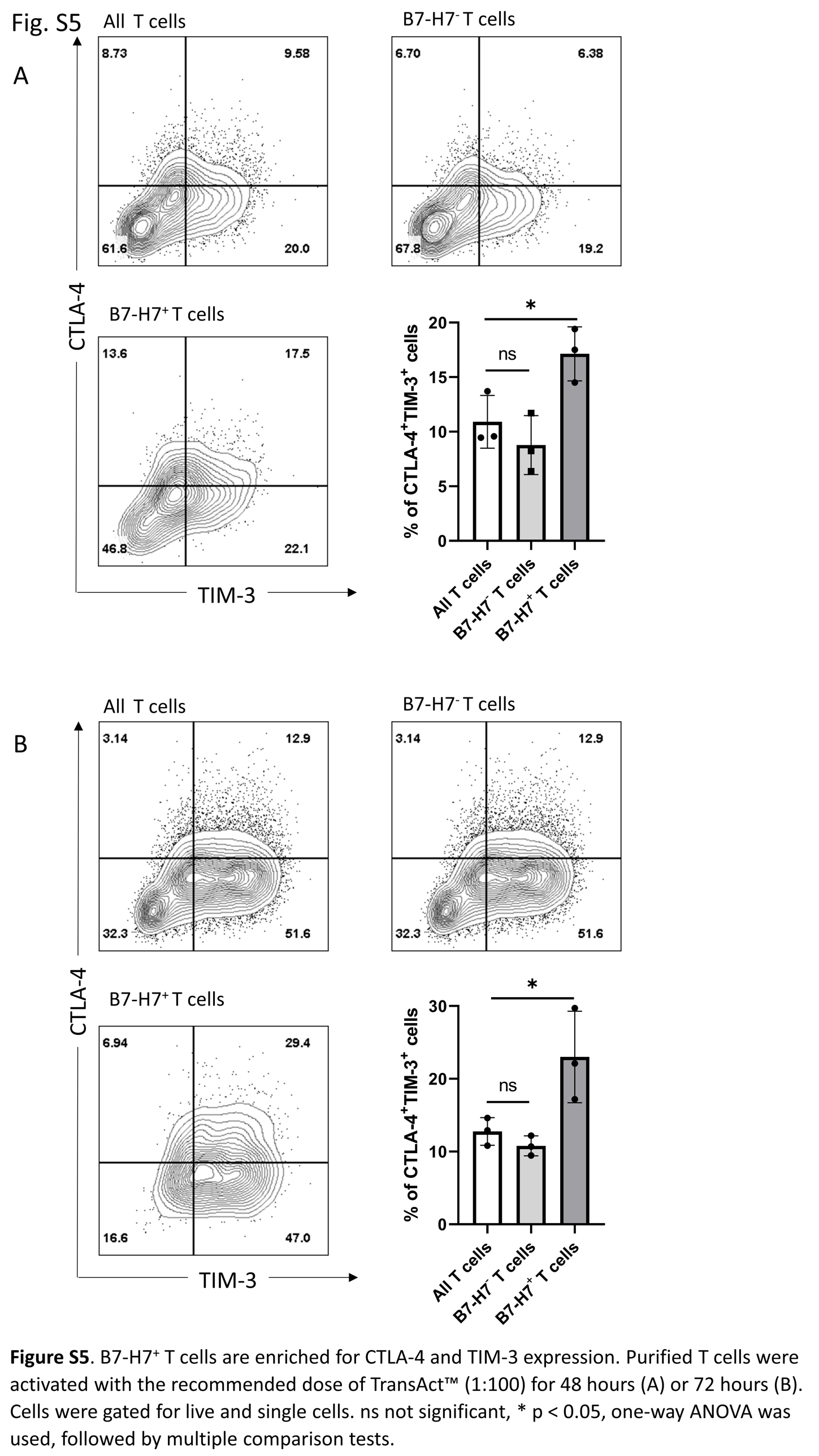

Supplement: Supplementary file 5 [file Image_4.jpeg]
